# Physicochemical Properties and Food Born Peptide Bioactivities of Fermented Cocoa Beans

**DOI:** 10.1155/tswj/5491217

**Published:** 2026-03-17

**Authors:** Winda Haliza, Endang Yuli Purwani, Dedi Fardiaz, Maggy Thenawidjaja Suhartono, I. Putu Wardana, Nur Laili, Diana Nur Afifah, Wayan Trisnawati

**Affiliations:** ^1^ Indonesian Center for Agricultural Postharvest Standard, Bogor, Indonesia; ^2^ Research Center for Food Technology and Processing, National Research and Innovation Agency (NRIA), Cibinong Science Center, Bogor, Indonesia; ^3^ Southeast Asian Food and Agricultural Science and Technology, SEAFAST Center, Bogor Agricultural University, Bogor, Indonesia, ipb.ac.id; ^4^ Research Center for Economic of Industry, Services, Trade, National Research and Innovation Agency (NRIA), Jakarta, Indonesia; ^5^ Departmen of Nutrition Science, Faculty of Medicine, Universitas Diponegoro, Semarang, Central Java, Indonesia, undip.ac.id; ^6^ Laboratory of Sustainable Diet and Biodiversity, Center of Research and Service–Diponegoro University (CORES-DU), Integrated Laboratory, Universitas Diponegoro, Semarang, Central Java, Indonesia, undip.ac.id

**Keywords:** angiotensin-converting enzyme, antioxidant, cocoad bean, fermentation, peptides

## Abstract

This study examined the physicochemical properties and bioactive peptides of fermented cocoa beans from the Indonesian regions of West Java, Yogyakarta, East Java, and Bali. Samples were analyzed for fermentation index, pH, free amino acids, total phenolic content (TPC), fat, and protein content. After the fat and polyphenol were removed, the remaining material was separated according to its solubility. The molecular weights were then evaluated using SDS‐PAGE. The ACE inhibitory activity was evaluated by the hippuryl‐L‐histidyl‐leucine (HHL) assay, and the antioxidant activity was analyzed by the di‐phenyl‐picryl‐hydrazine (DPPH) radical scavenging activity (DRSA) assay. All samples showed full fermentation (FI 1.0–1.6; pH 5.1–5.8) with various contents of TPC, fat, and protein. Free amino acid profiles from different regions were not significantly different. Identified protein fractions included albumin (21 kDa), globulin (31–45 kDa), prolamin (27 kDa), and glutelin (75–200 kDa). All fractions exhibited bioactivity, with albumin showing the strong antioxidant and ACE inhibitory effects. Albumin might act as an ACE inhibitory activity through competitive mechanisms, whereas other protein fractions might exert their effects through noncompetitive pathways. This study highlights the value of fermented cocoa for future product development.

## 1. Introduction

Cocoa plays a fundamental role for many nations in Africa and Asia, especially Indonesia, providing millions of smallholder farmers with a substantial income and contributing substantially to rural livelihoods and national economies. Globally, the cocoa industry supports an extensive value chain, from cultivation and processing to chocolate manufacturing and international trade. The production of premium cocoa beans relies on several processing procedures, including fermentation, drying, and roasting. Among these, fermentation is a critical stage for generating precursor compounds that drive the chemical and sensory quality of cocoa‐based products during roasting ([1, 2]). Traditionally, cocoa fermentation is carried out on‐farm by indigenous methods, driven by microbial activity that alters the bean composition and affects its physicochemical characteristics [3, 4].

The four components of cocoa beans′ protein fractions—albumin, globulin, prolamin, and glutelin—were abundantly available [5–8]. It undergoes intensive modification during fermentation. The information about proteolysis of cocoa protein involved in producing cocoa aroma precursors by endogenous enzymes was almost well understood [5]. In addition to their sensory role, protein‐derived peptides are increasingly recognized as bioactive compounds with potential health benefits, including antioxidant, antihypertensive, antihyperglycemic, antitumor, and antimicrobial activities [9–16].

Angiotensin converting enzyme (ACE) is a key regulator of hypertension, a major risk factor for cardiovascular disease, as it elevates blood pressure through the inactivation of vasodilatory bradykinins. The presence of ACE inhibitory peptides can mitigate this effect, thereby contributing to blood pressure reduction. Research has shown that cocoa peptides may suppress the activity of ACEs and act as antioxidants [17, 18]. However, most studies focus on cocoa from non‐Indonesian origins or on processed cocoa products, and there is a lack of data about bioactive peptides derived from fermented cocoa beans in Indonesia, particularly in relation to individual protein fractions.

Previous studies have not systematically compared the physicochemical properties, amino acid profiles, and bioactivities of protein fractions from Indonesian cocoa beans across different geographical origins. Moreover, data on the ACE inhibitory and antioxidant potential of these fractions—albumin, globulin, prolamin, and glutelin—from Indonesian fermented cocoa remain scarce.

This study characterizes the physicochemical properties, protein fractions, and bioactivities (antioxidant and ACE inhibitory) of fermented cocoa from four geographically distinct cocoa‐producing regions in Indonesian: Sukabumi Regency–West Java (approximately 6° 57 ^′^ S to 7° 25 ^′^ S and 106° 49 ^′^ E to ~107° 00 ^′^ E), Gunung Kidul Regency—Yogyakarta (approximately 7° 46 ^′^ S to 8° 09 ^′^ S and 110° 21 ^′^ E to 110° 50 ^′^ E), Jember Regency–East Java (approximately 7° 59 ^′^ 6 ^″^ S to 8° 33 ^′^ 56 ^″^ S and 113° 16 ^′^ 28 ^″^ E to 114° 03 ^′^ 42 ^″^ E) and Tabanan Regency–Bali (approximately 8° 14 ^′^ 30 ^″^ S to 8° 30 ^′^ 07 ^″^ S and 114° 54 ^′^ 52 ^″^ E to 115° 12 ^′^ 57 ^″^ E). These locations were selected because they represent established cocoa‐producing centers and offer clear geographical variation, thereby providing meaningful contrast for comparative analysis. The findings provide the first evidence that Indonesian cocoa is a potential source of bioactive peptides for functional food applications.

## 2. Materials and Methods

### 2.1. Chemicals

Analytical‐grade reagents were used throughout the study. ACE (0.1 U/mg of protein from rabbit lung), hippuryl‐L‐histidine‐L‐leucine (HHL), 2,2‐diphenyl‐1‐picrylhydrazyl (DPPH), and other chemicals were purchased from Sigma Chemical Co. (St. Louis, Missouri, United States).

### 2.2. Fermented Cocoa Samples

Bulk fermented cocoa beans were of the Forestero type, collected from four Indonesian regions: Sukabumi (West Java), Gunung Kidul (Yogyakarta), Jember (East Java), and Tabanan (Bali). The fermentation methods followed local farmers′ practices standardized by the Ministry of Agriculture. Approximately 40 kg of cocoa beans were fermented in perforated wooden boxes for 4–6 days, depending on the region, with regular turning to ensure uniformity. After fermentation, the beans were sun‐dried to a moisture content below 7.5% and stored at −20°C in plastic bags.

### 2.3. Preparation of Defatted Cocoa Powder and Acetone Dry Powder (AcDP)

Beans (500 g) were deshelled, ground, and initially defatted using n‐hexane. A second defatting step was performed using Soxtherm extraction. The final defatted powder was sieved (60 mesh). Defatted cocoa powder was further processed into AcDP to stabilize the storage protein [5]. Polyphenols were removed from defatted cocoa powder using successive extractions with 80% and 70% cold aqueous acetone, followed by dehydration using 100% acetone. The outcoming AcDP was stored at −20°C.

### 2.4. Fractionation of Cocoa Proteins [19]

The protein fraction in AcDP was obtained through a stepwise extraction. Albumin was extracted using Tris‐HCl with EDTA, globulin with a solution of NaCl, Tris‐HCl, and EDTA, prolamin with 70% ethanol, and glutelin with sodium hydroxide (NaOH). After extraction, each protein fraction was dialyzed at 4°C for 48 h and subsequently freeze‐dried. The concentration of proteins was determined using the BSA method.

#### 2.4.1. Determination of Physicochemical Characteristics

The fermentation index was determined following Apriyanto [20] using the absorbance ratio at 460/530 nm. The pH was measured after extracting 10 g of sample with 90 mL distilled water, heating (15 min), and filtering (Whatman No. 4). Total phenolic content was analyzed by the Folin–Ciocalteu method [21] and expressed as mg GAE/100 g.

#### 2.4.2. Determination of Free Amino Acids

Free amino acids were analyzed using the PICO TAG method with reverse‐phase HPLC (Waters, United States), equipped with a UV detector (254 nm) and a 3.9 × 150 − mm column. AcDP samples (0.1 g) were hydrolyzed in 6 N HCl at 100°C for 2 h, filtered, derivatized with methanol‐based reagents, and dried under vacuum. After adding sodium acetate buffer, samples were analyzed using a gradient elution with sodium acetate buffer (eluent A) and 60% acetonitrile (eluent B).

#### 2.4.3. SDS‐PAGE Analysis

Protein samples were precipitated with trichloroacetic acid (TCA), washed with cold acetone, dissolved in 2× Laemmli buffer containing 200 mM 2‐mercaptoethanol, and boiled for 5 min. SDS‐PAGE was carried out according to Voigt et al. [22], and gels were stained with Coomassie Brilliant Blue R‐250. Protein molecular weights were estimated using a 10‐250 kDa marker (Bio‐Rad).

#### 2.4.4. DPPH Radical Scavenging Activity (DRSA) Assay

DRSA was determined following the method of Tovar‐Pérez et al. [23]. Briefly, samples were mixed with 0.5 mL of 0.1‐mM DPPH in methanol, incubated in darkness at room temperature for 30 min, and shaken. The protein content of the sample was 0.03 mg/mL. Absorbance was measured at 517 nm using methanol as the blank and deionized water as the control. The percentage of DRSA was calculated using the following Equation (1):

DRSA %=Acontrol−AsampleAcontrol×100

#### 2.4.5. ACE Inhibitory Activity

ACE inhibitory activity was determined using a modified method of Sarmadi et al. [18]. Briefly, 100 *μ*L of cocoa fraction was mixed with 100 *μ*L of HHL (7.6 mM in 0.05 mM sodium borate buffer, pH 8.3, containing 0.4‐mM NaCl) and incubated at 37°C for 5 min. Subsequently, 50 *μ*L of ACE was added, and the mixture was incubated at 37°C for 1 h. The reaction was terminated by adding 250 *μ*L of 1 N HCl. Afterward, 1.5 mL of ethyl acetate was added, vortexed for 15 s, and centrifuged at 4000 × g for 10 min at 4°C. One milliliter of the ethyl acetate layer was evaporated at 95°C for 20 min, and the resulting hippuric acid was dissolved in 3 mL of 1 M NaCl. The sample contained 0.03 mg/mL of protein. Absorbance was measured at 228 nm. A control without cocoa fraction was prepared, and ACE inhibition was calculated using the following Equation (2):

ACE inhibition %=Acontrol−AsampleAcontrol×100

### 2.5. Statistical Analysis

All analysis was performed at least in triplicate. Analysis of variance (ANOVA) was performed to compare means and variances with a significant level of 95% (*p* < 0.05).

## 3. Results and Discussion

### 3.1. Physicochemical Characterizatics of the Fermented Cocoa

The physicochemical properties of the fermented cocoa samples, as shown in Table 1, reveal notable differences among regions. Fermentation causes polyphenols to migrate and undergo redox reactions, leading to a color change detectable at 460 nm. All samples met the fermentation index criteria (1.0–< 1.6) for well‐fermented beans [24]. The pH values aligned with previous studies [25]. Acid diffusion, mainly acetic acid, from pulp into cotyledons lowers pH and facilitates enzymatic reactions [26–28]. Varied TPC, fat, and protein content were also found among the samples. It was reported that TPC is a key indicator of cocoa quality [29]. The varied properties depend on the fermentation practices. At West Java and Jogjakarta, fermentation took place for 5 days, during which the beans were turned every day for the first 3 days. In East Java, the beans were turned daily during the 4‐day fermentation, whereas in Bali, fermentation extended to 6 days with turning every 2 days. The recorded local daily temperature during fermentation was 21°C–32.4°C. In addition, humidity and microbial activity across regions might influence the rate of biochemical reactions during fermentation.

**Table 1:** Physicochemical characteristics of fermented cocoa bean.

Characteristics	Fermented cocoa beans from			
	West Java	Yogyakarta	East Java	Bali
Fermentation index (FI)	1.25 ± 0.02^a^	1.26 ± 0.03^a^	1.48 ± 0.09^b^	1.53 ± 0.13^c^
pH	5.8 ± 0.02^b^	5.1 ± 0.04^a^	5.4 ± 0.02^a^	5.2 ± 0.01^a^
Phenolic content (mg GAE/g) ^∗^	129.52 ± 0.52^a^	157.52 ± 0.13^b^	196.75 ± 0.34^d^	187.95 ± 0.42^c^
Fat (g/100 g) ^∗^	44.75 ± 0.69^a^	45.56 ± 0.71^a^	46.08 ± 0.83^a^	47.41 ± 0.94^b^
Total protein *N* (g/100 g) ^∗^	17.18 ± 0.14^a^	17.73 ± 0.15^a^	15.35 ± 0.12^a^	15.15 ± 0.16^a^

*Note:* The  ^∗^ symbol denotes dry base. Data with the same letter in each row are not significantly different (*p* < 0.05). Reported values correspond to the mean ± standard deviation.

### 3.2. Profile of Free Amino Acids

Fermentation promotes enzymatic hydrolysis of cocoa proteins, releasing free amino acids (Table 2) that influence umami (Glu), sweet (Ala, Gly, Pro, Ser, Thr), and bitter (Arg, His, Ile, Leu, Met, Phe, Val) flavors [30, 31]. Flavor precursors, particularly Leu, Phe, and Ala, lead to aroma compounds such as 3‐methyl butanal and phenylacetaldehyde. Leucine and glucose contribute to the sweet chocolate aroma. Aspartic protease and carboxypeptidase release hydrophobic amino acids and peptides before proteolysis ends [32]. Glutamate and leucine, with potential antihypertensive effects, were found at levels comparable to fermented shrimp sauce [33]. Amino acid profiles varied by cocoa type, origin, and fermentation method [34].

**Table 2:** Free amino acids concentration (g 100 g^−1^) in fermented cocoa beans.

Fermented cocoa beans from				
Free amino	West Java	Yogyakarta	East Java	Bali
Asp^+^	1.65 ± 0.21^a^	1.56 ± 0.12^a^	1.49 ± 0.16^a^	1.51 ± 0.27^a^
Glu^+^	3.18 ± 0.78^a^	3.16 ± 0.23^a^	2.68 ± 0.49^a^	2.58 ± 0.16^a^
Ser	0.69 ± 0.10^a^	0.66 ± 0.14^a^	0.53 ± 0.23^a^	0.49 ± 0.17^a^
His ^∗^	0.51 ± 0.21^a^	0.42 ± 0.15^a^	0.34 ± 0.11^a^	0.48 ± 0.22^a^
Ala^++^	1.86 ± 0.15^a^	1.60 ± 0.02^a^	1.59 ± 0.23^a^	1.77 ± 0.36^a^
Tyr^++^	0.66 ± 0.32^a^	0.59 ± 0.31^a^	0.54 ± 0.11^a^	0.55 ± 0.32^a^
Val^++^	1.01 ± 0.20^a^	0.86 ± 0.28^a^	0.73 ± 0.25^a^	0.89 ± 0.44^a^
Ile	0.66 ± 0.42^a^	0.69 ± 0.35^a^	0.76 ± 0.43^a^	0.75 ± 0.43^a^
Leu^++^^∗^	1.82 ± 0.31^a^	1.87 ± 0.24^a^	1.73 ± 0.23^a^	1.75 ± 0.29^a^
Phe^++^^∗^	0.78 ± 0.25^a^	0.64 ± 0.21^a^	1.52 ± 0.19^a^	0.64 ± 0.29^a^
Gly	0.77 ± 0.22^a^	0.77 ± 0.02^a^	0.68 ± 0.11^a^	0.69 ± 0.22^a^
Arg	0.46 ± 0.08^a^	0.32 ± 0.02^a^	0.28 ± 0.04^a^	0.23 ± 0.04^a^
Thr ^∗^	0.79 ± 0.23^a^	0.61 ± 0.08^a^	0.54 ± 0.08^a^	0.60 ± 0.23^a^
Pro	0.65 ± 0.15^a^	0.69 ± 0.22^a^	0.53 ± 0.17^a^	0.51 ± 0.23^a^
Met	0.31 ± 0.01^a^	0.21 ± 0.07^a^	0.56 ± 0.21^a^	0.49 ± 0.21^a^
Cys	0.28 ± 0.11^a^	0.35 ± 0.09^a^	0.30 ± 0.08^a^	0.27 ± 0.08^a^
Lys ^∗^	0.81 ± 0.2^a^	0.77 ± 0.18^a^	0.81 ± 0.26^a^	0.78 ± 0.32^a^
Total	16.88	15.75	15.58	14.96

*Note:* ^+^: acidic amino acid; ^++^: hydrophobic amino acid;  ^∗^: essential amino acid. Data with the same letter in each raw are not significantly different (*p* < 0.05). Reported values correspond to the mean ± standard deviation.

### 3.3. Profile of Cocoa Protein Fractions

**Figure 1:**
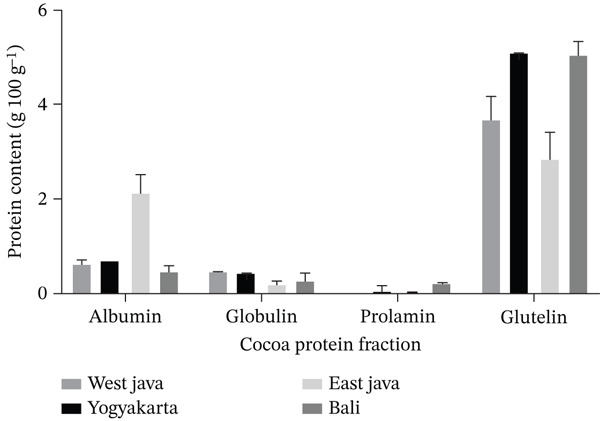
Protein fraction content in fermented cocoa beans of different origin. Value corresponds to the mean ± standard deviation D (*n* = 3).

**Figure 2:**
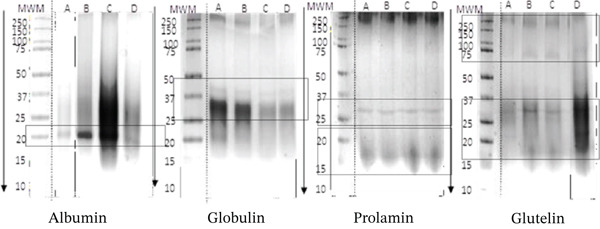
SDS‐PAGE profiles of cocoa protein fractions. Line A: West Java; B: Yogyakarta; C: East Java; D: Bali.

Proteins from AcDP powder were fractionated into albumin, globulin, prolamin, and glutelin. As shown in Figure 1, glutelin was the dominant fraction (75%–84%), followed by albumin (11%–14%), globulin (3%–9%), and prolamin (0.3%–6%). Variations in protein distribution across studies may result from differences in cocoa variety, extraction methods, fermentation, and storage. Albumin and globulin degradation during fermentation produces aroma precursors [5, 35]. SDS‐PAGE analysis (Figure 2) revealed similar band patterns across samples, with varying intensities. Albumin showed a dominant 21‐kDa polypeptide, globulin had bands at 45 and 31–37 kDa, and prolamin showed a 27‐kDa band. Glutelin displayed major bands at 200 and 75 kDa, with minor bands at 19.7, 47, and 31–37 kDa, the latter two overlapping with globulin, consistent with earlier reports [5, 8, 19, 22, 23].

### 3.4. Antioxidant Activity and ACE Inhibitory Activities of Fraction

All cocoa protein fractions showed antioxidant (Table 3) and ACE inhibitory activity (Table 4). As polyphenols were removed during sample preparation, the antioxidant effects were therefore provided by the peptides formed during fermentation. These peptides, particularly those containing the amino acids histidine, tyrosine, proline, alanine, and leucine, acted as electron donors to neutralize free radicals [36–38]. Despite being a minor fraction with relatively low molecular weight, albumin showed stronger DRSA than the predominant glutelin fraction. This suggests that the smaller peptide size in albumin enhances electron‐donating capacity, highlighting its potential as a key source of bioactive peptides in fermented cocoa. The results further demonstrated that the DRSA activity of fermented cocoa was higher than that reported for other fermented products, including soy‐based foods [38].

**Table 3:** Antiaoxidant activity (%) of protein fraction.

Protein fraction	Fermeneted cocoa from			
	West Java	Yogjakarta	East Java	Bali
Albumin	15.78 ± 2.81^a^	91.16 ± 1.61^c^	50.16 ± 4.64^b^	66.96 ± 2.71^b^
Globulin	5.27 ± 0.3^a^	9.35 ± 0.71^a^	30.25 ± 1.18^ab^	55.76 ± 0.31^b^
Prolamin	9.69 ± 2.94^ab^	5.22 ± 0.16^ab^	12.09 ± 0.02^a^	7.10 ± 2.10^b^
Glutelin	2.47 ± 0.3^ab^	0.86 ± 0.01^a^	1.07 ± 0.07^a^	3.43 ± 0.58^b^

*Note:*Data with the same letter in each raw are not significantly different (*p* < 0.05). Reported values correspond to the mean ± standard deviation. The protein concentration of tested samples was 0.03 mg/mL.

**Table 4:** ACE inhibitory activity (%) of protein fraction.

Protein fraction	Fermented cocoa from			
	West Java	Yogjakarta	East Java	Bali
Albumin	17.48 ± 3.11^a^	89.74 ± 1.59^c^	58.45 ± 5.20^b^	65.02 ± 2.63^b^
Globulin	18.91 ± 1.22^b^	6.54 ± 0.50^c^	14.37 ± 0.56^a^	16.70 ± 0.09^b^
Prolamin	16.18 ± 8.58^b^	4.83 ± 0.15^a^	1.01 ± 0.00^a^	0.10 ± 0.03^a^
Glutelin	1.99 ± 0.29^a^	1.66 ± 0.01^a^	2.92 ± 0.19^ab^	4.43 ± 0.75^b^

*Note:* Data with the same letter in each raw are not significantly different (*p* < 0.05). Reported values correspond to the mean ± standard deviation. The protein concentration of tested samples was 0.03 mg/mL.

The albumin‐derived peptide fraction from West Java cocoa showed the lowest antioxidant activity and ACE inhibitor activity relative to samples from other regions. Variations in environmental conditions and microbial communities may have restricted the enzymatic and microbial breakdown of the albumin fraction, thereby limiting the release of bioactive peptides. Because the antioxidant and ACE‐inhibitory activities of cocoa peptides are closely associated with the extent of proteolysis and the resulting peptide profile, these combined factors offer a plausible explanation for the markedly reduced antioxidant activity observed in the West Java albumin fraction.

**Figure 3:**
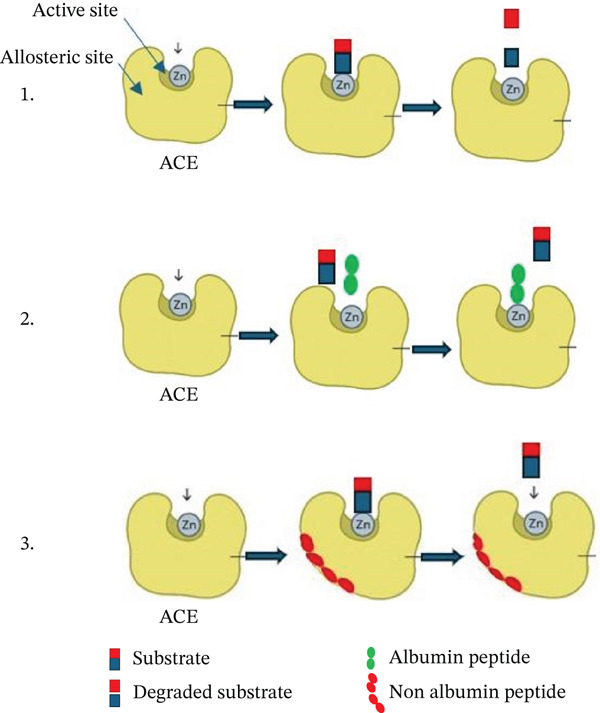
ACE inhibition mechanisms by protein fractions.

Moreover, the albumin fraction exhibited stronger ACE inhibitory activity than the more abundant glutelin fraction. This indicates that multiple mechanisms may be involved in the inhibition process. It is possible that albumin acts as a competitive inhibitor by vying with the synthetic substrate HHL for access to the enzyme′s active site, thereby blocking substrate hydrolysis. This interpretation aligns with previous reports indicating that low molecular–weight, hydrophobic peptides preferentially interact with the ACE catalytic pocket and typically exert competitive or competitive‐like modes of inhibition [39–41]. Given that ACE is a zinc‐dependent metalloprotease, albumin might also interfere with the Zn^2+^ ion, disrupting its coordination and impairing enzymatic function. In contrast, protein fractions with higher molecular weights than albumin likely exert noncompetitive inhibition by binding to allosteric sites, triggering conformational changes that diminish enzyme activity. The inhibitory action of the peptide against ACE was deeply reviewed [42]. Variations in structure and solubility among the fractions may account for these distinct inhibitory mechanisms [41]. These proposed mechanisms are illustrated in Figure 3.

## 4. Conclusions

Fermented cocoa beans from four Indonesian regions were fully fermented, with glutelin and albumin identified as the major protein fractions. Albumin showed the strongest antioxidant and ACE inhibitory activities, particularly in the Yogyakarta sample. These results suggest distinct inhibitory mechanisms among protein fractions and underscore the potential of Indonesian fermented cocoa as a source of bioactive peptides for functional food applications.

## Author Contributions

Winda Haliza: conceptualization, formal analysis, data curation, writing – original draft, funding acquisition. Maggy Thenawidjaja Suhartono: conceptualization, methodology, resources, supervision, writing – review and editing. Dedi Fardiaz: conceptualization, methodology, supervision, writing – review and editing. Endang Yuli Purwani: conceptualization, resources, writing – review and editing, supervision. I. Putu Wardana: writing – review and editing, funding acquisition. Nur Laili: writing – review and editing, project administration. Diana Nur Afifah: writing – review and editing, funding acquisition. Wayan Trisnawati: writing – review and editing, visualization.

## Funding

This study was supported by the Indonesian Agency for Agricultural Research and Development (104/Kpts/KP.440/1/2014), National Agency for Research and Innovation (6/III.12/HK/2024) and World Class University Program of Diponegoro University (229/UN7.A/HK/IV/2024).

## Disclosure

Endang Yuli Purwani has overall responsibility for the manuscript.

## Conflicts of Interest

The authors declare no conflicts of interest.

## Data Availability

The data that support the findings of this study are available from the corresponding author upon reasonable request.
